# News Stance Discrimination Based on a Heterogeneous Network of Social Background Information Fusion

**DOI:** 10.3390/e25010078

**Published:** 2022-12-30

**Authors:** Yanze Ren, Yan Liu, Jing Chen, Xiaoyu Guo, Junyu Shi, Mengmeng Jia

**Affiliations:** 1 Henan Key Laboratory of Cyberspace Situation Awareness, Zhengzhou 450001, China; 2 Linyi Vocational College, Linyi 276016, China

**Keywords:** news stance discrimination, heterogeneous network, social background information fusion, multiple attribute information, node feature representation

## Abstract

Media with partisan tendencies publish news articles to support their preferred political parties to guide the direction of public opinion. Therefore, discovering political bias in news texts has important practical significance for national election prediction and public opinion management. Some biased news often has obscure expressions and ambiguous writing styles. By bypassing the language model, the accuracy of methods that rely on news semantic information for position discrimination is low. This manuscript proposes a news standpoint discrimination method based on social background information fusion heterogeneous network. This method expands the judgment ability of creators and topics on news standpoints from external information and fine-grained topics based on news semantics. Multi-attribute features of nodes enrich the feature representation of nodes, and joint representation of heterogeneous networks can reduce the dependence of position discrimination on the news semantic information. To effectively deal with the position discrimination of new news, the design of a multi-attribute fusion heterogeneous network is extended to inductive learning, avoiding the cost of model training caused by recomposition. Based on the Allsides dataset, this manuscript expands the information of its creator’s social background and compares the model for discriminating political positions based on news content. In the experiment, the best transductive attribute fusion heterogeneous network achieved an accuracy of 92.24% and a macro F1 value of 92.05%. The effect is improved based purely on semantic information for position discrimination, which proves the effectiveness of the model design.

## 1. Introduction

**Figure 1:**
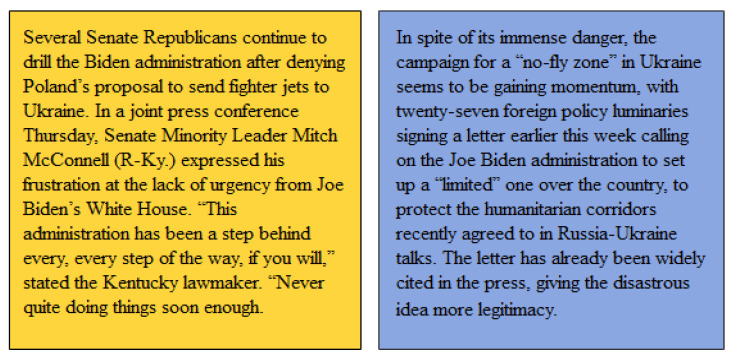
An example of differences in news positions in “Russian–Ukrainian military conflict”.

In the past decade, we have witnessed intensification of civic discourse and increasing polarization of political ideology. As the Associated Press put it, “Americans are more divided than ever, deadlocked on social issues, race, gender, and the economy.” This polarization trend also applies to the rest of the world. Political stance has become the dominant factor in daily activities, and we live in an era of echo rooms and party misinformation. These unprecedented situations require researchers to strengthen identification of political stances. Today, news released by the media is widely spread on social networks, accelerating the impact on people’s political views [1]. For example, the two political news articles shown in Figure 1, respectively, describe the left-leaning and right-leaning political attitudes of the United States towards the event of the “Russian–Ukrainian military conflict”. Right-leaning news shows more frustration with Biden’s policies; left-leaning news shows support for Biden’s legislation. Some media support their political parties by publishing biased news, guiding the masses’ ability to judge the “Russia–Ukraine conflict”, changing their political views, and paving the way for the next election. Therefore, it is very important to select traditional news without political bias and enable the audience to objectively and fairly evaluate current affairs.

**Figure 2:**
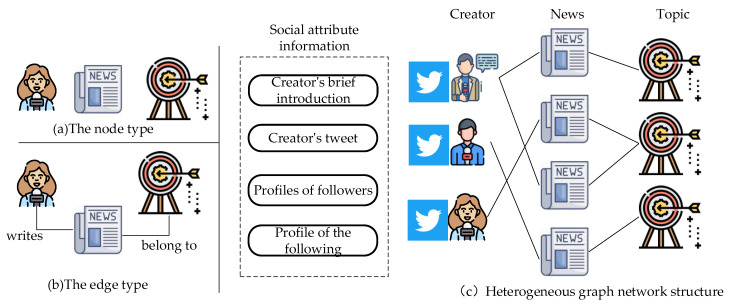
An example of a heterogeneous network with social background information fusion.

Traditional judgment of political stance mostly starts from the content, but it is difficult to detect the bias in the news. News creators often express their positions obscurely, which prevents the traditional language model from being able to identify their political stance correctly [2]. Social network communication of news and the entity background knowledge map involved in the news are closely related to political views. These characteristics ensure that news position detection should not only remain in its content. We can excavate more useful information from different angles to enhance the judgment of the news stance [3]. Some works have introduced various external information, including selecting entities in the news to introduce the existing knowledge graph and constructed political knowledge graph for position discrimination or relying on spread of news in social media for news position discrimination [4]. This paper still starts from the social network, relies on the relationship between the news and the author, and introduces the author’s rich social background information to distinguish the news stance. For example, the creator of a news piece will show his political stance when he writes the news, and his related remarks and relationships on social media can also reflect his political stance. At the same time, the news topic can increase the news’s relevance. Therefore, collecting relevant information is meaningful in judging the news’s political bias. To make more effective use of the above information, we combine news, topic, and author information and model them as heterogeneous networks with a strong ability to express rich information. In this work, we model the problem of news political stance discrimination as the classification of news nodes in heterogeneous networks. Figure 2a includes the type of nodes appearing in the graph, Figure 2b includes the type of edges in the graph, and Figure 2c describes the heterogeneous network instance in this scenario.

In order to address the above challenges, this paper proposes a method of discriminating news stances by relying on the author’s social background information and constructs a new two-level attention heterogeneous network model that can fuse multi-attribute information to solve such problems. The multi-attribute information of coded nodes is then fused. For each news article node, two-level attention is adopted. The optimal combination of different types of neighbors can be obtained hierarchically. The learned nodes represent features captured from different heterogeneous information sources. Through backpropagation, the model can be optimized end-to-end to obtain the optimal feature representation of news nodes, and the node classification task is used to distinguish the political stance of news. In addition, the model is extended to inductive learning to identify the categories of new nodes in the heterogeneous network. The inductive graph is constructed through the newly emerged news, and the trained model is used as the aggregation function to learn the features of nodes on the inductive graph. The inductive learning method of heterogeneous networks avoids retraining the model every time to cope with the new nodes and reduces the calculation overhead. In summary, this work makes the following contributions:

- As far as we know, this is the first attempt to encode the semantic features of the creator’s social background multi-attribute information and extract the graph’s structural features to represent each node’s features.
- This work constructs a heterogeneous network, including news, topics, and creators. A two-layer attention heterogeneous network model with multi-attribute characteristics of fusion nodes is designed. The multi-attribute features of nodes are fused, and the feature representation is updated through the heterogeneous network. The designed model has comprehensive evaluation ability when the effective feature attributes cannot be selected. It can still realize the ability of high-precision news position discrimination.
- The dual-level attention heterogeneous network model with multi-attribute aggregation is used as the aggregation function of inductive learning. The existing labeled data are used to train the model, and the node categories on the constructed inductive graph are determined. High-precision identification of news position is realized, and the cost of model retraining caused by new nodes is reduced.

## 2. Related Work

In this section, we first briefly introduce the research status of political news discrimination and understand various modeling methods. Then, the research progress of heterogeneous neural networks is introduced briefly, which provides the knowledge foundation for establishment of a heterogeneous network model of news social background.

### 2.1. Research Status Quo of Discriminating Political Stance of News

Similar to the text classification task, the research on position discrimination of news by extracting linguistic features from text includes: Ahmed et al. [5] and Bhatia et al. [6] model the ideological perspective at the thematic level to infer the political orientation of news, respectively. Iyyer et al. [7] and Chen et al. [8] both applied recurrent neural networks (RNN) to identify political tendencies at the sentence level. Li et al. [9] studied the political orientation of news texts from the semantic perspective and injected entity information into the text model to identify the differences in news narratives from different perspectives. Ren et al. [10] established a heterogeneous network by extracting the entities, topics, and sentences in the news, updated node representations through the heterogeneous network, and aggregated node features to make stand discrimination in the news.

Researchers that use various external information, including knowledge graphs and social network communication, to discriminate news stances include Chen et al. [11], who proposed a method to predict political orientation based on the opinion knowledge graph. The author integrated the views and target entities extracted from the text into the existing structured knowledge base to form the graph structure and carried out ideological reasoning through the information dissemination on the graph. Feng et al. [12] constructed an American political knowledge graph containing 10,703 triples to strengthen the representation of the political orientation of news. The author constructs a graph network of news content and identifies its political orientation by combining the map of small-scale domain knowledge. Li et al. [13] capture how news spreads in social networks and use graph convolution networks to capture the news’s social background to judge the news’s orientation. Li et al. [14] proposed a pre-trained model to make it easier for the model to detect news bias by using rich social background and language information, including entity mention and news sharing. Feng et al. [15] proposed entity detection of political positions and political ideology judgment of social entities. Baly et al. [16] combined multi-source news information to comprehensively judge the political orientation of news, that is, who wrote it, how it was described in social media, and who read it.

Unlike the above studies, we model the news from the topic and the author simultaneously and try to use the author’s multi-attribute social background information to mine the author’s potential political stance and further realize discrimination of the news stance.

### 2.2. Development and Application of Heterogeneous Graph Neural Network Model

Heterogeneous networks can deal with the problems of different nodes and edges in graph networks. They aggregate the information of different types of nodes, making each node fuse more information. In the study of the heterogeneous network model, HAN [17] used semantic-level attention and node-level attention to learn the importance of meta-path and node neighbors simultaneously and obtained the final node representation through corresponding aggregation operations. Unlike HAN, HetGNN [18] does not consider node-level attention but uses LSTM to aggregate the node neighbors in a certain relationship and update the node representation. Its neighbor selection is also different, and a fixed number of neighbors are selected by random walk restart. Ling et al. [19] proposed CoarSAS2hvec. To better collect rich information in heterogeneous networks, the HIN coding program self-avoiding short-sequence sampling (CoarSAS) was used, and the optimized loss function was used to improve the system’s performance. Heterogeneous networks are also used in many scenarios. Hu et al. [20] created the HGAT model to classify short texts, extract entities and topics from texts to construct heterogeneous networks, and capture their rich relationships to solve the problem of semantic sparsity of short texts. Hu et al. [21] created a CompareNet model to extract information from news, construct a heterogeneous network, and compare it with Wikipedia knowledge to detect fake news. Ren et al. [22] constructed a heterogeneous network of news creators and topics and designed a heterogeneous information network with hierarchical attention for fake news detection. Various heterogeneous network models have been proposed, and the improvement and innovation have been relatively complete. At the same time, they have been applied to short text classification, fake news detection, and recommendation systems. However, they have almost no application in news standpoint discrimination. In terms of related ideas, a heterogeneous network architecture is designed to represent news node features and further discriminate news positions.

## 3. Method

### 3.1. Problem Definition

This experiment uses political news dataset, which comes from major news media websites. The data are universal. The social background information is obtained through the real name author information, and the heterogeneous network is constructed by combining the subject information of news. The specific concepts are defined as follows:

News stance classification: Compared with the current popular fake news detection work, stance discrimination has a more complex dimension. There are many manifestations of journalistic stance bias. D’Alessio and Allen reviewed 59 quantitative studies on partisan media bias in presidential elections and defined journalistic stance bias as following three forms based on their findings: (1) Gatekeeping bias is where editors and reporters “choose” which stories to cover. (2) Coverage bias is the amount of news (such as the length of newspaper articles or television airtime) received by a social group that is purposefully biased towards one side at the expense of the other. (3) State bias means that news media insert their attitudes or opinions into news reports [23]. In the American political context, position bias is classified as left-leaning, neutral, and right-leaning through different manifestations, with left-leaning people more likely to be Democrats and right-leaning people more likely to be Republicans in terms of their party performance.

Social context multi-attribute heterogeneous networks are defined as follows: for a network G(V,E,A,C,R), V represents the set of nodes in the network, E represents the set of edges in the network, A represents the set of attributes in the network, C represents the set of node types, and R represents the set of edge types. The set V contains |V| nodes, and, for any node v∈V, there is a mapping ψ that makes ψ(v)=c, c∈C, |C| is the number of node types. |E| is the number of edges, and, for any edge e∈E, there is a mapping Ω that makes Ω(e)=r, r∈R; |R| is the number of edge types. Set A contains |A| attributes, and all attributes include two types: node attributes and relationship attributes. For any node attribute attv∈A, there is a mapping Φ(attv)∈V; that is, every node attribute must belong to a node and will not exist alone. For any relational attribute atte∈A, there exists a mapping X(atte)∈E; that is, every relational attribute must belong to some edge and again will not exist alone. If |C|+|R|>2, the network is G defined as a heterogeneous network. If A≠ϕ, then the network G is defined as an attribute network. Network G is defined as a multi-attribute heterogeneous network if |C|+|R|>2 and A≠ϕ.

Node classification of news heterogeneous network: The heterogeneous news network with social background is described as G. For the constructed news N, creator C, and topic T, we connect all data nodes into the following heterogeneous network diagram G=(V,E), where V represents the set of nodes, V={N,C,T}. E represents the set of edges. Multiple creators complete a news article. Here, a link between the creator and the news is established, and a news article will be published under a certain theme. Therefore, the edge connection between news and theme is established, and the set of edge is finally obtained as E={(N−C)∪(N−T)}.After construction of the social background of multi-attribute heterogeneous network, application of double attention heterogeneous networks with multiple attribute information fusion model occurs, as well as the news political stance discriminant model for the node classification task. The input of this model is multiple attribute information of heterogeneous network nodes, and rich representation of news graph can be learned through the characteristics of heterogeneous network model. At the same time, in order to accommodate the computational overhead caused by the recomposition of new nodes, we design an inductive graph neural network model, which aims to learn the characteristic representation of nodes rather than the fixed values. According to our task, we defined the stance of news as left-leaning, neutral, and right-leaning, namely y∈{0,1,2}.

### 3.2. Model Design

News position can be not only reflected by its content but also by the creator’s position, which is highly consistent with the news he writes. How to obtain the creator’s political stance more accurately plays an important role in judging the political stance of the news. Therefore, we should consider how to select and obtain the multi-attribute social background information of the creator to represent the characteristics of the creator node. At the same time, we need to put forward a general heterogeneous network model with the function of attribute information fusion and be able to carry out attribute information fusion and dimension expansion. Finally, the appropriate graph network structure is used to transfer the creator node information to the news node, which makes the news feature representation of the same political stance closer.

**Figure 3:**
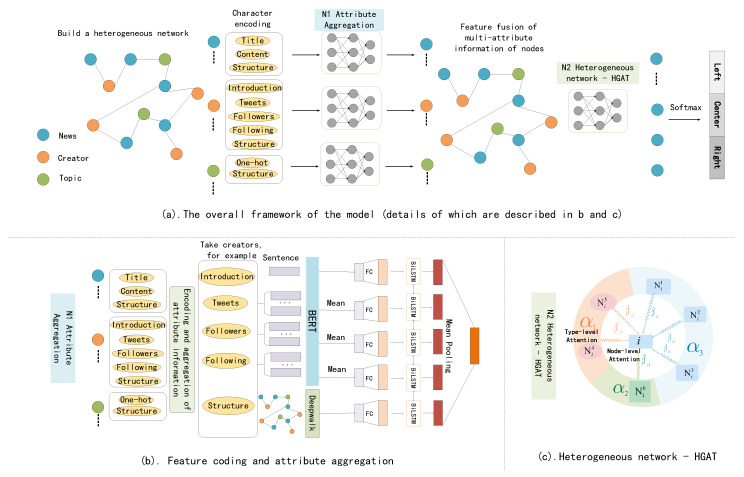
The overall framework of news stance discrimination based on a heterogeneous network of social background information fusion.

In this manuscript, the overall framework of the model is shown in Figure 3a. Figure 3b,c includes the details of the model. An end-to-end learning process is designed to build a heterogeneous network for all news, creators, and topics. The overall framework is specifically divided into the following parts: (1) construct a heterogeneous network containing the multi-attribute information of nodes; (2) aggregate the multi-attribute information of a single node to obtain node feature vectors; (3) use the two-layer attention heterogeneous network model to aggregate node features; (4) obtain the final feature representation of news nodes, and then classify the political stance of news.

#### 3.2.1. Construction of a Multi-Attribute Heterogeneous Network

This manuscript selects the nodes related to news stances to construct a heterogeneous network and designs multi-attribute information for each node type.

Structural attribute features: to make the nodes rich in a certain range of structural characteristics, this manuscript uses the random walk sampling method for the constructed heterogeneous network, samples a certain length sequence of nodes, and then uses the Word2vec coding sequence to obtain the feature vector representation of each node and obtains the node feature code as the structural attribute characteristics of each class of nodes.

News: the set of news nodes is described as N, which does not rely solely on news content. Therefore, we obtain the codes of news nodes $N_{Title}=\left\{n_{1},n_{2},\cdots ,n_{m}\right\}$, $N_{Content}=\left\{o_{1},o_{2},\cdots ,o_{m}\right\}$, $N_{\mathrm{Deepwalk}}=\left\{ns_{1},ns_{2},\cdots ,ns_{m}\right\}$ through the coding of news title, content, and structure attributes, where m represents the number of news items, and n, o, and ns represent the corresponding three attribute codes.

Creator: the set of Creator nodes is described as C. Each article contains one or more creators. There are n creators in all news collections. To obtain the feature representation of creators, we introduce creators to the social platform to obtain the multi-attribute information of creators and obtain their profiles on social platforms. The feature representation of each creator is $C_{desc}=\left\{d_{1},d_{2},\cdots ,d_{n}\right\}$, and the feature representation is $C_{tweets}=\left\{w_{1},w_{2},\cdots ,w_{n}\right\}$. $C_{following}=\left\{fg_{1},fg_{2},\cdots ,fg_{n}\right\}$ is the feature obtained from the profile of a follower’s friend list. The profile of the followers’ friends list is represented as $C_{followed}=\left\{fd_{1},fd_{2},\cdots ,fd_{n}\right\}$. The structural attribute of the creator is characterized by $C_{\mathrm{Deepwalk}}=\left\{cs_{1},cs_{2},\cdots ,cs_{n}\right\}$. The above n represents the number of nodes of the creator. d,w,fg,fd,cs is the feature code corresponding to the above five attributes of the creator.

Topic: the topic node is described as T, and the published news is often classified into a certain topic. The topic can establish the relevance between news, and the corresponding feature representation is $\left\{t_{1},t_{2},\cdots ,t_{v}\right\}$. The structure attribute characteristic of the topic is $T_{\mathrm{Deepwalk}}=\left\{ts_{1},ts_{2},\cdots ,ts_{v}\right\}$. v represents the number of news topic nodes, and t,ts are the two attribute feature codes corresponding to the topic nodes.

**Figure 4:**
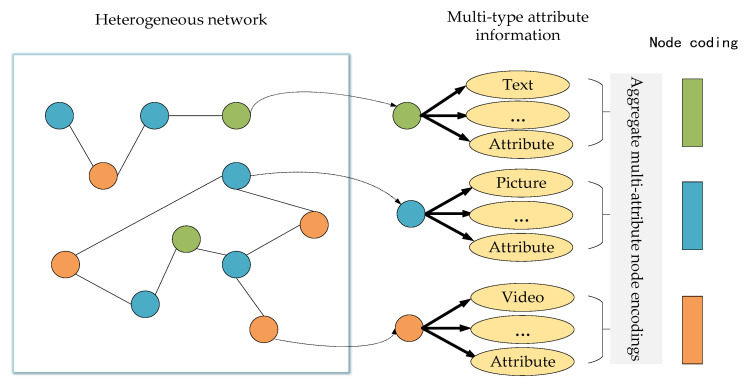
Coding and aggregation of node multi-attribute information.

For the different attributes of the above types of nodes, the corresponding coding method is used to obtain the feature representation of the corresponding attributes. The general block diagram is shown in Figure 4 below.

For a given node’s attribute information, it adopts different encoding methods according to its types, such as text and picture. However, it tries to show its position characteristics as much as possible. The node structure attribute set in this manuscript obtains the node structure feature through DeepWalk, and other social and news content attributes are text type, so Bert is selected as its coding. Because Bert has been trained by much information, it already has the shallow features of the text.

#### 3.2.2. Multi-Attribute Information Aggregation Strategy Based on Bi-LSTM

In the case of nodes with multi-attribute coding, the simple use of means, splicing, and other methods will lead to physically meaningless features, and the obtained features weaken the previous single attribute features. Therefore, Bi-LSTM is used as a multi-attribute aggregation method. The advantages of this method include: 1. The complexity is relatively low, so it is relatively easy to implement the model and adjust parameters. 2. Different from directly splicing different types of content features into a vector, the model uses Bi-LSTM to capture deep feature interaction, which can fuse heterogeneous content information and obtain stronger representation ability. 3. It is flexible to add additional content features, and the model is easy to expand.

Using Bi-LSTM to aggregate attribute information [18], the following formula calculates the multi-attribute aggregate feature representation of each node *v*:

(1) $$f_{1}\left(\upsilon \right)=\frac{\sum_{i\in C_{\upsilon }}\left[\vec{LSTM}\{ℱC_{\theta_{x}}\left(x_{i}\right)\}⊕\overset{\leftarrow }{LSTM}\{ℱC_{\theta_{x}}\left(x_{i}\right)\}\right]}{C_{\upsilon }}$$

$f_{1}\left(\upsilon \right)$ is the final vector representation containing multi-attribute aggregation.$ℱC_{\theta_{x}}$ is a feature transformation layer, which can be identity transformation and a full connection layer to transform different content features. The content of each attribute is encoded as $x_{i}$, which is aggregated by Bi-LSTM and averaged according to the number of attributes; $C_{\upsilon }$ represents the multi-attribute of a node. Bi-LSTM operates on an unordered multi-attribute set $C_{\upsilon }$. Its inspiration comes from previous work on aggregating unordered neighbors [24]. We use different Bi-LSTM to aggregate the content characteristics of different types of nodes because their content is different from each other.

#### 3.2.3. Convolution Algorithm for Bilevel Attention Heterogeneous Networks

We use the heterogeneous graph neural network to deal with the heterogeneous graph. The heterogeneous graph neural network model can deal with different types of nodes and different types of edges in the graph. Considering the difference between various types of information, for example, for news nodes, the author neighbor and the topic neighbor have different degrees of influence on news. In addition, famous creators and ordinary authors have different influences on the news. Therefore, this work uses a heterogeneous network convolution algorithm with double attention [25]. They are projected into an implicit common space by their respective transformation matrices.

The calculation method is as follows: the $H^{\left(l+1\right)}$ representation of the heterogeneous convolution l+1 layer is updated by aggregating different neighbor nodes $H_{\tau }^{l}$; the initial node vector matrix is $H^{\left(0\right)}=X$, the rows of X are all nodes, and the columns are the characteristics of all nodes. The specific formula is as follows:

(2) $$H^{\left(l+1\right)}=\sigma (\sum_{\tau \in T}{ℬ}_{\tau }\cdot H_{\tau }^{\left(l\right)}\cdot W_{\tau }^{\left(l+1\right)})$$

In the formula, σ(⋅) represents the activation function, and node τ has a different transition matrix $W_{\tau }^{\left(l\right)}$. The transition matrix $W_{\tau }^{\left(l\right)}$ considers different feature spaces and projects them into the same feature space. ${ℬ}_{\tau }\in R^{\left|\upsilon \right|\times \left|\upsilon_{\tau }\right|}$ represents the attention shift matrix, the row represents all nodes, and the column corresponds to its neighbor nodes under the τ type. The specific element value in the matrix ${ℬ}_{\tau }$, such as ν row and $\nu^{\prime }$ column value $\beta_{\nu \nu^{\prime }}$ is calculated as follows:

(3) $$\beta_{\nu \nu^{\prime }}=Softmax_{\nu^{\prime }}\left(\sigma \left(\nu^{T}\cdot \alpha_{\tau }\left[h_{\nu },h_{\nu^{\prime }}\right]\right)\right)$$

ν is the attention vector, and $\alpha_{\tau }$ is the attention weight of different types of nodes. $h_{v}$ and $h_{\nu^{\prime }}$ represent the current node vector and the vector of its adjacent node $\nu^{\prime }$, respectively. The Softmax function is used for normalization across adjacent nodes of node ν.

In the aggregation of node information, the calculation method of attention weight $\alpha_{\tau }$ of different types of nodes is as follows:

(4) $$\alpha_{\tau }=Softmax_{\tau }\left(\sigma \left(\mu_{\tau }{}^{T}\cdot \left[h_{\nu },h_{\tau }\right]\right)\right)$$

where the Softmax function is used to normalize across all types, $\mu_{\tau }$ is the attention weight under τ type, $h_{\nu }$ is the embedded representation of the current node, $h_{\tau }$ is the sum of the weight of all neighbor nodes $h_{\nu^{\prime }}$ under type τ. The above σ(⋅) is the activation function, and Leaky Relu is used specifically.

Through the L-layer heterogeneous graph convolution network, we can obtain the news node vector representation that combines the news’s rich semantic information and the creators’ social background information.

#### 3.2.4. Inductive Learning of Heterogeneous Graph Neural Networks

In practical applications, the news will be updated constantly generated over time. Adding new nodes to the graph each time means that the features of many related nodes will be updated, and the graph network model needs to be retrained in the reconstructed graph containing all nodes, which leads to great computational overhead. For this purpose, an inductive heterogeneous graph neural network model is designed. The purpose of this model is to learn the representation of a node rather than to obtain the characteristic representation of the node. When distinguishing the position of a newly added news node, we construct a new heterogeneous graph including the node. In this section, the convolution algorithm of the double-layer attention heterogeneous network described in Section 3.2.3 is used as the aggregation function of nodes. This algorithm can fully consider the differences between nodes and edges, and the aggregation model trained in the original heterogeneous network is applied to the new heterogeneous network subgraph for the feature representation of the newly added nodes and the discrimination of news stance [25].

**Figure 5:**
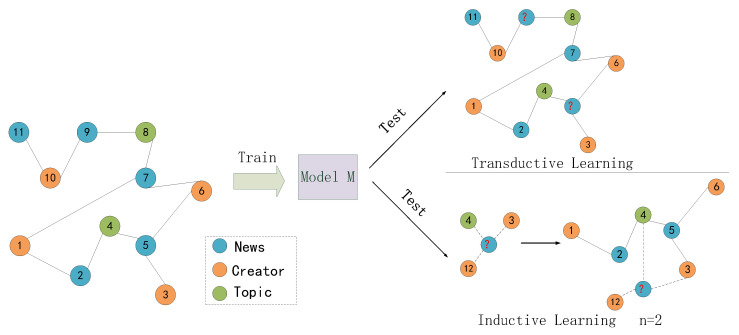
Inductive learning of heterogeneous graph neural network.

The specific method of inductive learning of the heterogeneous graph neural network can be described as shown in Figure 5. First, a graph network G=(V,E) is constructed by relying on the existing training and testing data, and the aggregation function is obtained by relying on the graph training. A new heterogeneous network $G_{new}=\left(V_{new},E_{new}\right)$ is established according to its author and topic information for a newly added news node. Nodes that are the same as $V_{new}$ and V (i.e., nodes 3 and 4 in the figure) are used for N-hop neighbor expansion. The extended nodes and news nodes to be judged are added to the heterogeneous network $G_{new}=\left(V_{new},E_{new}\right)$ to obtain the extended inductive graph $G^{\prime }=\left(V^{\prime },E^{\prime }\right)$.

#### 3.2.5. Model Training

After fusion of multi-attribute two-layer heterogeneous network in L-layer, we can obtain the final embedded representation $H^{\left(L\right)}$ of nodes in the network, then transport the embedded expression to Softmax for classification, and use the news article node with a label y to train the classifier; the classification results are three kinds of results of news political stance y∈{0,1,2}, which are expressed as follows:

(5) $$Z=Softmax\left(H^{\left(L\right)}\right)$$

In model training, we use the L2 norm to train the cross-entropy loss function of data.

(6) $$L=-\sum_{i\in D_{train}}\sum_{j=1}^{C}Y_{\mathrm{ij}}{\log(Z}_{\mathrm{ij}})+\eta {\left\|\Theta \right\|}_{2}$$

where C is the number of categories, $D_{train}$ is the index of news items in the training set, $Y_{\mathrm{ij}}$ is the label matrix corresponding to the political stance of news. Θ is the model parameter and η is the regularization factor. For model optimization, we use a gradient descent algorithm.

## 4. Experiment

### 4.1. Experimental Setup

#### 4.1.1. Dataset Description

Our experiment is based on the Allsides news dataset. The relevant description of the dataset is as follows: the data source is the Allsides website, which will push news articles from all aspects of the political spectrum for each hot event. The website will hide the source of the articles and provide the final value according to the reader’s evaluation of the political tendency of the articles. In this experiment, Baly et al. [26] used the large-scale news dataset created by this website to publish 37,554 news articles from 73 news media obtained from this website, covering more than 100 topics. The news represents real political scenes. It should be noted that the intermediate category covers articles biased towards centrist political ideology rather than those lacking political bias (such as sports and technology). Table 1 shows the statistical results of the dataset.

Most of the above datasets contain the creator’s information, and the news with the creator’s information is retained. At the same time, the creator’s name is linked to the social platform for retrieval. The social platform recommends some user information for us according to the relevance of the query. We crawled all the profiles of the creators on the platform, the comments they posted, the profiles of the followers, and the profiles of the people they followed. We cleaned the recommended wrong data, screened out more than 17,000 news items for the experiment, and used the social background information of the creators corresponding to these news items as the social background dataset of the experiment.

#### 4.1.2. Experimental Setup and Evaluation Method

In this manuscript, we use the python deep learning framework as the experimental development environment, the deep learning server uses an NVIDIA GV100 graphics card, and all text information is encoded using Bert-base-uncased. The encoder has 12 hidden layers, outputs 768-dimensional tensors, 12 self-attention heads, and a total of 110 m parameters obtained by training in small English text. Attribute aggregation uses Bi-LSTM. Other related parameters are set in Table 2.

The node attribute information is encoded as follows:

Structure attribute, the constructed graph network is encoded by DeepWalk, each node repeats 20 times, and the length of the walking sequence is 30. Then Word2Vec is used to obtain the structural attribute feature encoding of each category node.

Creator, using Bert to code profiles on their social platforms, the Bert model inserts a [CLS] symbol before the text. The output vector corresponding to the symbol is taken as the semantic representation of the whole text. The output obtains a 768-dimensional feature vector, which is used for the feature representation of the profile. At the same time, each published statement released by the creator is encoded in the same way, and then the average is taken to obtain the 768-dimensional feature vector of the attribute dimension. The profile information of multiple followers and the profile information of followers agree to take the average method after Bert coding to obtain the feature vector of the corresponding attribute.

News, using the title and content as the news feature, Bert encodes the title and content of the news, respectively, and the feature vectors on the two attributes of the news are obtained.

Topics, for 108 topics, use One-Hot to encode and expand the dimension to 768 dimensions. This node can add the correlation degree between news.

Evaluation method: political tendency was classified into three categories. Accuracy and macro F1 were used as evaluation indexes of practical effect. Accuracy refers to the proportion of correctly classified samples to the total number of samples. The F1 value is an index used to measure the accuracy of the classification model in statistics. It takes into account the accuracy and recall rate of the classification model. Macro F1 is to calculate the F1 of each category and then average it.

### 4.2. Experimental Results

In this work, all news, creators, and topics are constructed into a heterogeneous graph network to establish the correlation between news and news. The unknown news nodes can be inferred through the news with known labels. At the same time, this manuscript selects the creator node and provides the creator and news multi-attribute information. By coding the single attribute and fusing the multi-attribute information, we can obtain the information of the unknown news node. The effectiveness of this method is proven.

#### 4.2.1. Influence of Node Attributes on Experimental Results

**Figure 6:**
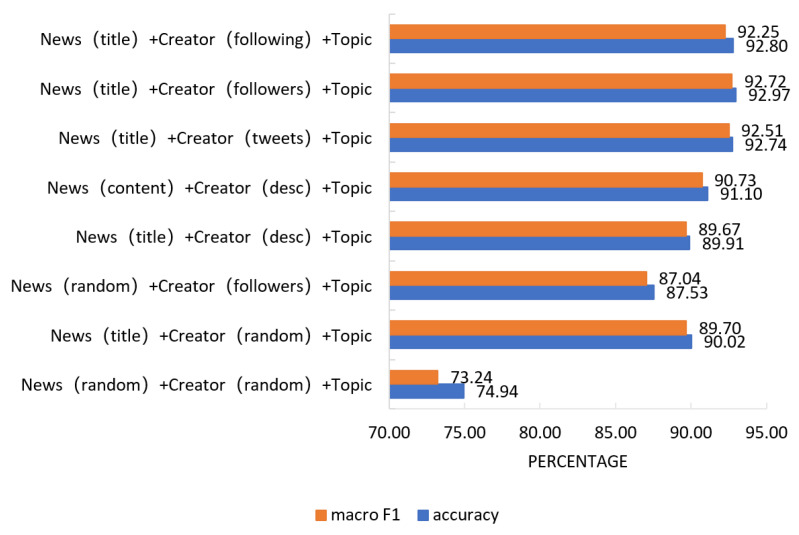
Influence of node attributes on experimental results.

In this experiment, the single attribute features of nodes are used in the constructed heterogeneous network. The three attributes defined in Section 3.1 are used by news, and the creators use the five attributes defined in Section 3.1. In order to prove the effectiveness of the node attributes selected in this paper, random coding was used to eliminate attribute information. The partition ratio of the 8:1:1 training set, validation set, and test set was used to study the importance of attributes by taking direct inference learning as an example, and experimental results, as shown in Figure 6, were obtained. The results show that accuracy of 74.94% and value of 73.24% macro F1 can be achieved using only heterogeneous networks without any attribute information injected. In the experiment with the single attribute information of news or the single attribute information of the creator, the experimental results are greatly improved, indicating that the attribute features we selected can improve the discriminant accuracy of news stances to a certain extent.

In addition, we controlled the relevant variables and compared the importance of each attribute information. We found that the representation effect of news content was higher than that of news titles, and the effect of the speech published by the creator and the profile information of followers was higher than that of the creator. It may be because the effect of a single data representation of the creator’s profile is not sufficient. Combined with multiple statements and profiles of multiple followers and followers, the standpoint characteristics of the node can be more fully expressed.

#### 4.2.2. Effect of Different Hop Counts on Experimental Results in Inductive Learning

**Figure 7:**
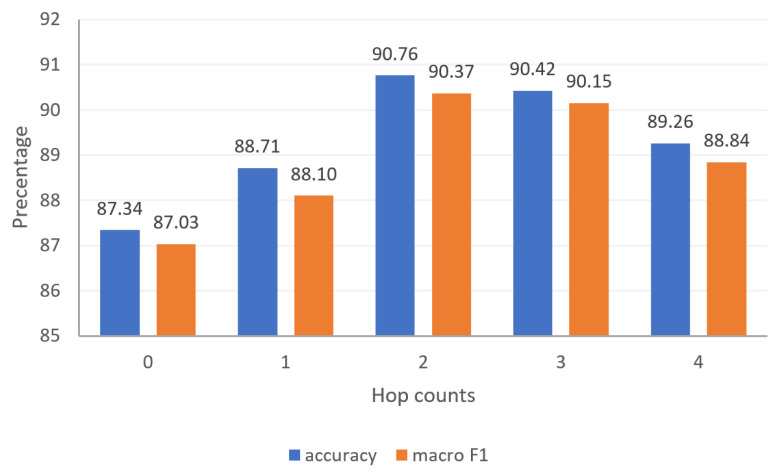
Influence of different hop counts for inductive learning on experimental results.

This section discusses the influence of inductive graphs constructed with different hop counts on the experimental results. The Figure 7 shows the accuracy and macro F1 value of inductive graphs constructed with hop counts from 0 to 4 for discriminating the stance node of news nodes. It can be seen from the results that the effect is poor when the hop count is 0 because the original label data are not used. Nevertheless, the representation of node characteristics can still help to judge test set node categories. The increase in hops improves the effect because the constructed inductive graph uses the existing label news data information. The gradual decrease in the number of hops starting from 3 is due to linking too many noisy nodes, affecting the feature representation of the central node.

#### 4.2.3. Comparative Analysis of Attribute Fusion Methods

**Figure 8:**
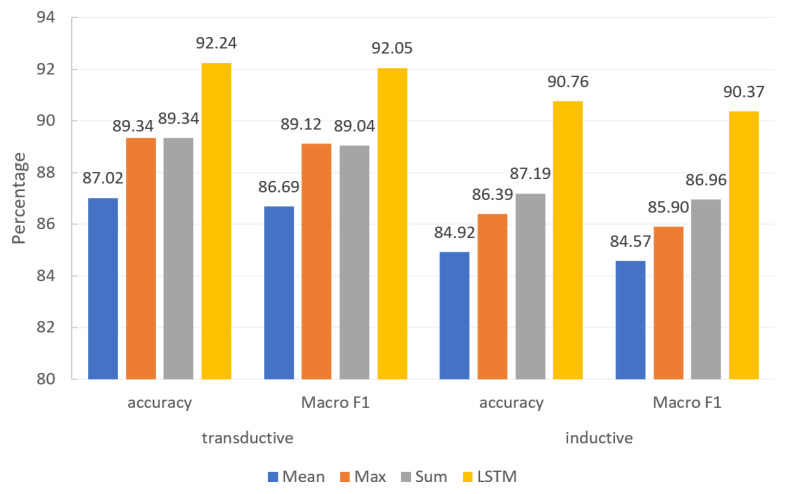
Effect of node attribute fusion method on experimental results.

In order to verify the effectiveness of the attribute fusion method designed in Section 3.2.2, the three attribute features of the news node are fused, and the five attribute features of the creator node are fused. The fusion strategy is to take the average of the multi-attribute features of a single node, take the maximum feature value, sum the feature value, and aggregate the multi-attribute information through Bi-LSTM. The experimental results shown in Figure 8 are obtained for transductive and inductive learning, respectively.

Through the experimental results, it is found that the result value is lower than that of single attribute information simply by averaging and maximum values of various attributes. Because the angles of each attribute description are different, the above calculation will lead to no practical meaning. The node characteristics cannot be described from any angle, so the original attribute characteristics of nodes are eliminated. The results are not as good as the single attribute features. The fusion strategy used in this manuscript is almost the same as the optimal value under the condition of a single attribute. For nodes with more attributes, selecting the attribute with characteristics is not easy. When it is considered that the importance of attributes cannot be judged, then the attributes can be fused and still achieve an optimal result.

#### 4.2.4. Influence of Labeled Data Proportion on Experimental Results in Transductive Learning

**Figure 9:**
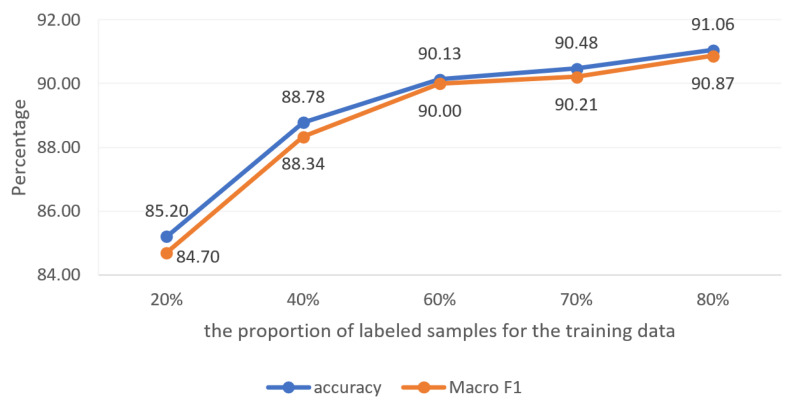
Influence of labeled data proportion on experimental results.

In this section, the multi-attribute features of all nodes are fused and used as the final feature vector to construct a heterogeneous network graph. This manuscript will study the influence of the proportion of news stance tags on the experimental results. The fixed test sample size remains unchanged, and the proportion of labeled samples for the training data is set as 20%, 40%, 60%, 70%, and 80%, respectively. The experimental results are shown in Figure 9. It can be seen that the coding method of fusing multi-attribute features designed in this manuscript can well represent the position characteristics of news nodes. Through fusion of heterogeneous networks, the data of labeled nodes are effectively used to enhance the feature representation of associated nodes. At the same time, in the case of only small sample data labels, relying on this feature representation method and heterogeneous network model, better classification results can still be obtained in the transductive learning network.

#### 4.2.5. Comparison with the Baseline Model

For the text classification model, we compare the model of this experiment with several baseline methods that use better text information processing effects, including the following methods. The experimental comparison results are shown in Table 3.

FastText [27]: The input is multiple words and their n-gram features representing a single document. The document vector is obtained by averaging the words and n-gram vector of the whole document, and then the document vector is used for classification.

TextCNN [28]: The convolutional neural network CNN is applied to the text classification task. Multiple convolution kernels of different sizes extract the key information in sentences to better capture the local correlation.

Bert [29]: The structure of the Transformer is adopted. The BERT model aims to use the large-scale unlabeled corpus to train and obtain the representation of text containing rich semantic information. The pre-training process is carried out by using the MASK of the word and the prediction of the next sentence on a large corpus. The Finetune process through the Bert model is suitable for a variety of text tasks.

ERNIE [30]: It aims to learn language representation enhanced by a knowledge masking strategy. Unlike Bert, the masking strategy includes entity masking and phrase masking and implicitly learns information about knowledge and long semantic dependencies to guide word embedding learning.

Ren et al. [10]: The heterogeneous network is established by extracting the entities, topics, and sentences in the news, and the node information is updated through the heterogeneous network. The aggregation of node information is taken as the feature representation of the news to make the newsstand discrimination.

FastText and TextCNN model parameters were set for experimental parameter setting: batch size = 128, pad size = 512, the learning rate was set as 1 × 10^−4^, and the hidden layer dimension was 256. The batch size of Bert and ERNIE is set as 32, pad size = 512, the learning rate is set as 5 × 10^−5^, and the hidden layer dimension is set as 768. Bert + CNN adds a convolution layer on Bert’s output; the convolution kernel size is (2,3,4), and the number of convolution kernels is 256. The model parameters used by Ren et al. ‘s method are: the number of topics is set as 3, the learning rate is set as 1 × 10^−5^, epochs = 50, and other model parameters are the same as the original paper.

After fusing social background information, this experiment achieved 92.24% accuracy and 92.05% macro F1 value. Generally, models such as FastText and TextCNN obtain feature representation of the sentence through modeling, while representation of political orientation is relatively complex. It will be impossible to distinguish the difference in political orientation, and the experimental results will be poor because of the simple reliance on the semantic information of the sentence. Bert, ERNIE, and Bert + CNN have a better effect because they use the generation method of dynamic word vectors to adjust the word vector representation according to the semantic information of the context. At the same time, Ren et al. model the heterogeneous network of content, which enables them to learn the subtle political bias in the news. The experimental results of the method in this paper are greatly improved, on the one hand, because features represent all the news that needs to be discriminated, and the creator node and topic node are used to model the news into a heterogeneous network, which enhances the information interaction between news. On the other hand, adding the creator’s multi-attribute social background knowledge can further improve the effect. The experimental results show that this modeling method is very effective for political orientation discrimination.

## 5. Conclusions

This paper proposes a heterogeneous graph neural network model based on multi-attribute social context information fusion for news political stance discrimination. The model constructs news with creators and topics into a heterogeneous network, generates a multi-dimensional social background information dataset of creators, and designs a multi-attribute fusion method of nodes. The multi-attribute features of the creator node, news node, and topic node are fused. The feature representation of the news node is updated through the heterogeneous network model. When effective node features cannot be selected, the maximum feature representation of node features is realized. High-precision stance discrimination of news nodes is realized. In addition, to deal with the stance discrimination of new news nodes, the designed model is extended to inductive learning, and a high accuracy discrimination effect is also achieved, proving this method’s effectiveness.

In future work, we will continue to work on two aspects. One is to design more nodes related to news stance discrimination to build a graph network model for enriching news stance detection. At the same time, considering using more node attribute information, we can adopt a better attribute fusion strategy to optimize the feature representation of nodes. The other is to consider extensible application of the model in this paper. The attribute fusion method can affect the knowledge graph [31], biomedicine, and other fields.

## Figures and Tables

**Table 1:** Data statistics.

	Political Complexion	Number of News	Proportion
**Allsides**	Tilt left (0)	13,005	34.6%
	Neutral (1)	10,815	28.8%
	Tilt right (2)	13,734	36.6%

**Table 2:** Parameter settings.

Hyperparameter	Value
Aggregation mode	Bi-LSTM
LSTM hidden layer dimension	384
Number of layers of LSTM stack	1
Heterogeneous network hidden layer dimension	512
Regularization factor η	5 × 10^−6^
Learning rate	1 × 10^−3^
dropout	0.5
epochs	2000
optimizer	Adam

**Table 3:** Comparison of baseline model experiment results.

Model	Accuracy	Macro F1
FastText	64.12	63.29
TextCNN	69.73	69.64
Bert	82.22	81.90
ERNIE	79.31	78.17
Bert + CNN	81.18	81.40
Ren et al. [10]	82.69	82.64
**Ours inductive**	**90.76**	**90.37**
**Ours transductive**	**92.24**	**92.05**

## Data Availability

Not applicable.
